# Endothelial Hyper-Permeability Induced by T1D Sera Can be Reversed by iNOS Inactivation

**DOI:** 10.3390/ijms21082798

**Published:** 2020-04-17

**Authors:** Alessandra Cazzaniga, Roberta Scrimieri, Elisa Giani, Gian Vincenzo Zuccotti, Jeanette A. M. Maier

**Affiliations:** 1 Department of Biomedical and Clinical Sciences “Luigi Sacco”, Università di Milano, 20157 Milan, Italy; roberta.scrimieri@unimi.it (R.S.); patgen@unimi.it (G.V.Z.); jeanette.maier@unimi.it (J.A.M.M.); 2 Humanitas Clinical and Research Center, 20089 Rozzano, Italy; generalepatologia@gmail.com

**Keywords:** endothelial cells, permeability, diabetes mellitus type 1, nitric oxide

## Abstract

Type 1 Diabetes Mellitus (T1D) is associated with accelerated atherosclerosis that is responsible for high morbidity and mortality. Endothelial hyperpermeability, a feature of endothelial dysfunction, is an early step of atherogenesis since it favours intimal lipid uptake. Therefore, we tested endothelial leakage by loading the sera from T1D patients onto cultured human endothelial cells and found it increased by hyperglycaemic sera. These results were phenocopied in endothelial cells cultured in a medium containing high concentrations of glucose, which activates inducible nitric oxide synthase with a consequent increase of nitric oxide. Inhibition of the enzyme prevented high glucose-induced hyperpermeability, thus pointing to nitric oxide as the mediator involved in altering the endothelial barrier function. Since nitric oxide is much higher in sera from hyperglycaemic than normoglycaemic T1D patients, and the inhibition of inducible nitric oxide synthase prevents sera-dependent increased endothelial permeability, this enzyme might represent a promising biochemical marker to be monitored in T1D patients to predict alterations of the vascular wall, eventually promoting intimal lipid accumulation.

## 1. Introduction

Type 1 Diabetes Mellitus (T1D) is a multifactorial disease characterized by chronic hyperglycaemia that arises from a T cell-mediated autoimmune attack of the pancreatic β-cells and culminates with the suppression of insulin production [1]. The worldwide incidence is rising by 3% per year and the major risk of mortality is due to cardiovascular complications caused by accelerated atherosclerosis [2,3]. Since the endothelium is the gate-keeper of vascular health, it is not surprising that endothelial dysfunction is the early event leading to the development of atherosclerotic lesions [4]. Non-laminar flow, metabolic challenge and inflammatory cytokines model endothelial function [5].In particular,, hyperglycaemia contributes to macrovascular endothelial dysfunction in T1D by activating multiple pathways through the accumulation of free radicals and glycolytic intermediates, among others [6]. Accordingly, a high percentage of paediatric patients with T1D shows endothelial dysfunction [7] strictly associated with poor glycaemic control. However, the mechanisms underlying the insurgence of cardiovascular damage in patients with T1D are not entirely known and, more importantly, there are no biomarkers for an early diagnosis. In vivo, endothelial dysfunction is defined by the inability of the artery to sufficiently dilate in response to a transient reduction of flow and is considered an indirect measure of nitric oxide (NO) released by the endothelium [8]. Considering in vivo, endothelial dysfunction is defined by altered NO synthesis, increased expression of inflammatory molecules, exaggerated generation of reactive oxygen species (ROS) and enhanced permeability of the cell layer [9]. 

NO is a simple molecule with complex biological activities. It is implicated in the regulation of many physiological pathways and it is a key player of vascular health, contributing to the maintenance of vascular tone and exerting anti-platelet, anti-thrombotic and anti-inflammatory properties [10]. Under physiological conditions, NO generated by endothelial NO Synthase (eNOS) represents the main source of circulating NO [11]. However, NO can be produced in excess in several clinical conditions, such as inflammation, when inducible NOS (iNOS) is activated by inflammatory stimuli. While low levels of NO are beneficial to harmonize coagulation, inflammation and vascular tone, high levels of NO exert detrimental effects, among which is a reversible increase of endothelial permeability [12], a relevant early event in atherogenesis.

In vivo NO is oxidized to the stable NO products nitrate and nitrite (NOx). A causal relationship between NO and plasma levels of NOx exists to the point that NOx plasma measurements reflect NO bioavailability [13]. Circulating high levels of NO have been reported in T1D patients [14,15] as well as in diabetic rats [16]. A recent meta-analysis discloses a significant increase in NOx levels in European T1D patients [17]. Interestingly, the high concentration of NO observed in the serum of paediatric T1D patients is responsible for mediating hyperfiltration and persistent microalbuminuria, thus linking vascular to glomerular dysfunction in hyperglycaemic conditions [14]. The increment of NO could be due to the induction of iNOS in phagocytes in response to the release of pro-inflammatory cytokines [15]. Moreover, an iNOS-induced elevation of circulating NO seems to be strictly correlated with insulin-resistant states [18].

On these bases, we measured NOx levels in the sera from 36 T1D paediatric patients and 14 healthy controls. Next, we evaluated the effects of these sera on endothelial permeability. 

## 2. Results

### 2.1. Serum NOx Levels and Endothelial Permeability Are Not Associated with Increased Glycated Haemoglobin in T1D Subjects

**Figure 1:**
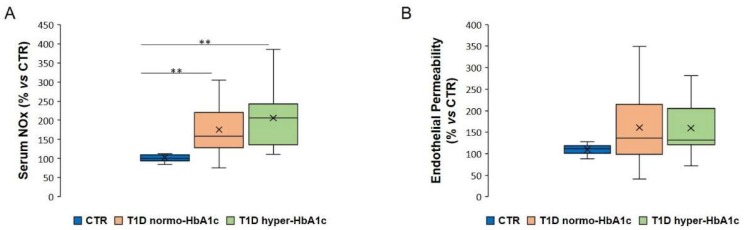
Amounts of NOx in the sera from healthy individuals, T1D patients with normal or high HbA1c and the effects of the same sera on endothelial permeability. The sera of patients were grouped according to the levels of HbA1c and their effects on HUVEC were compared to those of sera from healthy controls. (**A**) The levels of NOx were measured in the sera from healthy donors (CTR) and T1D subjects with normal or high HbA1c (T1D normo-HbA1c or T1D hyper-HbA1c). (**B**) The effect of the above-described sera on endothelial permeability was evaluated using a Transwell Permeability Assay. The results are the mean of three experiments in triplicate. ** *p* < 0.01.

Since the half-life of NO in the circulation is shorter than 0.1 s, circulating NO metabolites are assessed as indicators of NO production [13]. We utilized the Griess assay, a method widely used in epidemiologic studies [19], to measure NOx in the serum from healthy and T1D subjects. We initially grouped the sera from our T1D patients according to normal or high levels of glycated haemoglobin (HbA1c), the surrogate biochemical marker of the average glycaemia over a preceding period of 2–3 months. We detected statistically significant higher amounts of NOx in the sera from T1D patients, independently from the levels of HbA1c (Figure 1A). We then cultured HUVEC with the same sera (10%) for 24 h to test the endothelial barrier function and found a feeble, albeit not significant, increase of endothelial permeability in cells incubated with sera from T1D patients with normal or high HbA1c (Figure 1B).

### 2.2. Serum NOx Levels and Endothelial Permeability Are Associated with Hyperglycaemia

**Figure 2:**
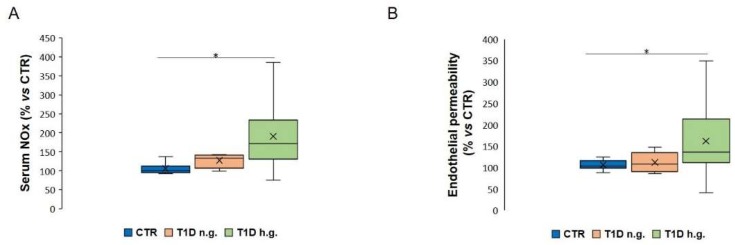
Determination of NOx in the sera from healthy individuals, T1D patients with normal or high glycaemia and effects of these sera on HUVEC permeability. The sera of patients were grouped according to fasting glycaemia. (**A**) The levels of NOx were measured in the sera from healthy subjects (CTR) and T1D individuals with normal (T1D n.g.) or high glycaemia (T1D h.g.) as described in the methods. (**B**) Endothelial permeability was measured in HUVEC exposed to 10% of the sera using a Transwell Permeability Assay. The results are the mean of three experiments in triplicate. * *p* < 0.05.

We then anticipated that the effects of the sera from T1D patients might depend upon high blood glucose. Therefore, we grouped these sera according to fasting glycaemia and compared the amounts of NOx in healthy, normo- and hyperglycaemic T1D subjects. Figure 2A shows that levels of NOx were significantly increased only in the sera obtained from hyperglycaemic subjects (T1D h.g.). The same result was obtained when we evaluated endothelial permeability in relation to glycaemia. Indeed, Figure 2B shows that permeability is markedly increased in HUVEC exposed for 24 h to sera from hyperglycaemic individuals (T1D h.g.), whereas no significant differences exist between sera from normoglycaemic T1D (T1D n.g.) and healthy subjects (CTR).

### 2.3. High Concentrations of Extracellular Glucose Increase Endothelial NOx Release and Permeability in Endothelial Cells 

**Figure 3:**
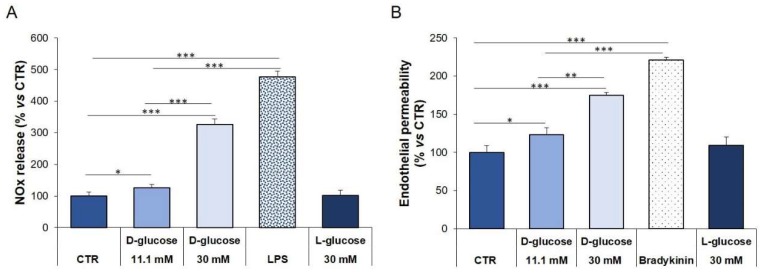
NOx release and permeability in HUVEC exposed to different concentrations of glucose. HUVEC were cultured in a medium containing 5 mM (CTR), 11.1 and 30 mM glucose for 24 h. LPS and Bradykinin were used as positive controls. (**A**) Media were collected and NOx levels were measured as described in the methods. (**B**) Endothelial permeability was studied as described in the methods. The results are the mean of three experiments in triplicates ± standard deviation (SD). * *p* < 0.05; ** *p* < 0.01; *** *p* < 0.001.

To get insights into a possible role of high glucose in inducing endothelial permeability, we performed experiments on HUVEC exposed to physiological (5.5 mM, CTR) or high (11.1 and 30 mM) concentrations of extracellular glucose for 24 h. Bradykinin (10 μM) was used as a positive control for endothelial permeability, while lipopolysaccharide (LPS, 10 μg/mL) was the positive control for NOx release. L-Glucose (30 mM) was utilized as a control of osmolarity. D-glucose increased endothelial release of NOx (Figure 3A) as well as permeability (Figure 3B) in a concentration-dependent manner, while L-glucose exerted no effects, thus indicating the pivotal role of high glucose, and not increased osmolarity, in inflecting endothelial performance.

### 2.4. The Upregulation of iNOS is Responsible for the Increase of NOx in HUVEC Exposed to High Glucose

**Figure 4:**
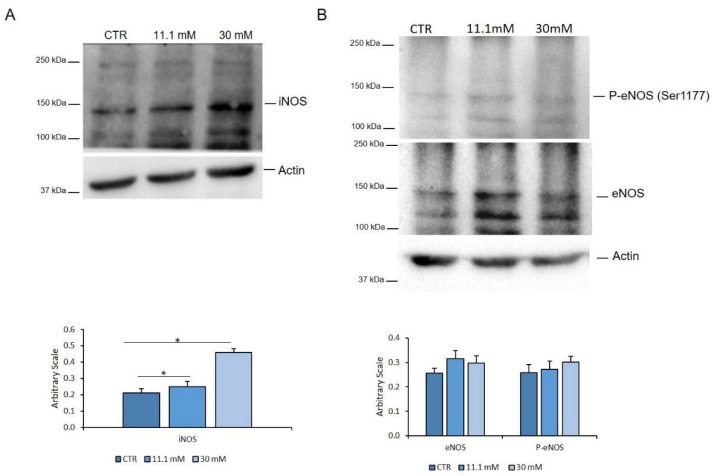
iNOS and eNOS in HUVEC exposed to different concentrations of glucose. HUVEC were cultured in a medium containing 5 mM (CTR), 11.1 and 30 mM glucose for 24 h. Western blot was performed using specific antibodies against iNOS (**A**), P-eNOS^Ser1177^, and eNOS (**B**). Actin was used as a marker of loading. The experiments were repeated three times and a representative blot is shown. Densitometry was performed by Image J software calculating the ratio between the protein of interest and actin on three separate experiments ± SD. * *p* < 0.05.

To understand which isoform of NOS is involved in the increase of NO upon treatment with high extracellular glucose, we assessed the total amounts of iNOS and eNOS, the two enzymes that catalyse the production of NO in endothelial cells. We also investigated the activated form eNOS, which is phosphorylated on Ser1177 (P-eNOS^Ser1177^). The total amount of iNOS were increased by high d-glucose (Figure 4A). Conversely, both the eNOS and P-eNOS^Ser1177^ were not significantly modulated by high glucose (Figure 4B).

**Figure 5:**
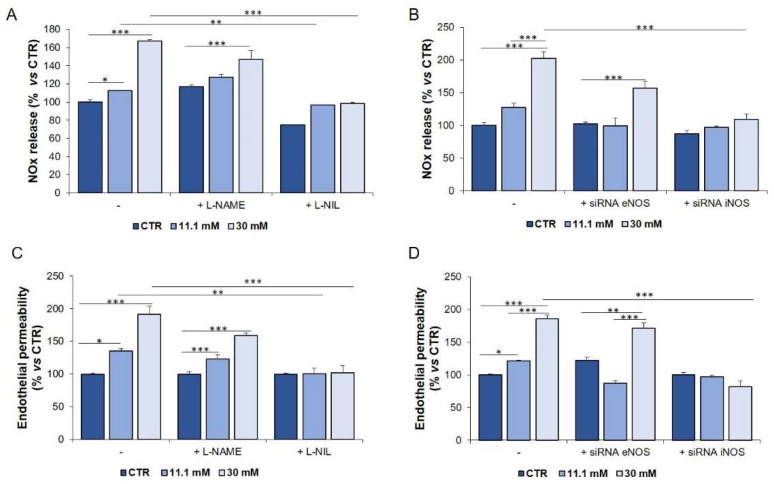
NOx release and permeability in HUVEC cultured in a medium containing different concentrations of glucose after genetic and pharmacological inhibition of iNOS or eNOS. HUVEC were cultured in a medium containing 5 mM (CTR), 11.1 and 30 mM glucose for 24 h in the presence of L-NAME or L-NIL (**A**,**C**) or after gene silencing (**B**,**D**). A scrambled non silencing sequence was used as a control (–) for silencing. The results are the mean of three experiments in triplicate ± SD. * *p* < 0.05; ** *p* < 0.01; *** *p* < 0.001.

We then assessed the role of iNOS and eNOS in modulating endothelial permeability. HUVEC were pre-treated for 1 h with L-NAME (100 µM) and L-NIL (100 µM), pharmacological inhibitors of eNOS and iNOS, respectively, and then exposed to a medium containing high concentrations of glucose for 24 h. In parallel, HUVEC were transiently transfected for 6 h with specific siRNAs targeting *iNOS* and *eNOS*, or a scrambled sequence as a control, and then exposed to high glucose for the following 24 h. Figure 5 shows that iNOS silencing as well as L-NIL prevented glucose-induced NOx accumulation and hyperpermeability, whereas L-NAME slightly reduced NOx release and permeability in HUVEC cultured in high glucose, as expected since eNOS is constitutively active in endothelial cells. 

### 2.5. Genetic and Pharmacological Inhibition of iNOS Restores Endothelial Permeability in Cells Exposed to Sera from Hyperglycaemic T1D Patients 

**Figure 6:**
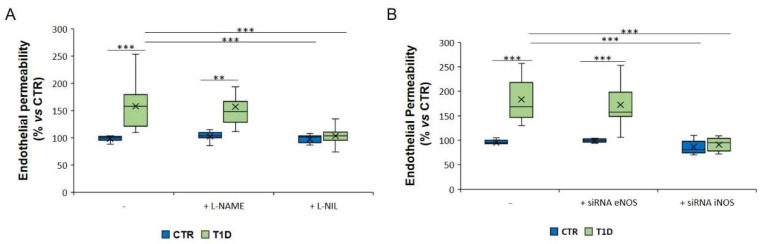
Permeability in HUVEC cultured in medium containing 10% hyperglycaemic T1D or CTR sera after genetic and pharmacological inhibition of iNOS or eNOS. HUVEC were cultured in the presence of sera from healthy and T1D subjects for 24 h with or without L-NAME or L-NIL (**A**) or after gene silencing (**B**). A scrambled non-silencing sequence was used as a control (–) for silencing. The results are the mean of three experiments in triplicate. ** *p* < 0.01; *** *p* < 0.001.

We then asked whether the increase of endothelial permeability by sera from hyperglycaemic T1D patients was dependent upon the induction of iNOS. Endothelial cells were pre-treated for 1 h with L-NAME (100 µM) or L-NIL (100 µM) before adding to the culture media 10% of T1D serum from hyperglycaemic or healthy subjects. As shown in Figure 6A, L-NIL reduced endothelial permeability to approximately the same level as the controls. We also transiently silenced *eNOS* and *iNOS* utilizing specific siRNAs (Figure 6B), while the controls were exposed to a scrambled sequence. We found a marked reduction of endothelial permeability when a siRNA was used against *iNOS*.

## 3. Discussion

T1D predisposes to premature atherosclerosis, the main reason for high morbidity and impaired life expectancy in T1D patients [20]. One of the earliest events in atherogenesis is the elevated permeability of the endothelium, which favours the accumulation of lipoproteins into the intima where they are oxidized and propagate endothelial dysfunction [4].

Here we show that sera from hyperglycaemic T1D subjects markedly enhance endothelial leakage, whereas sera from normoglycaemic T1D or healthy individuals do not. Since it suffices to expose HUVEC to high extracellular D-glucose to augment their permeability, we anticipate that hyperglycaemia is responsible for the increase of endothelial permeability observed when we use sera from hyperglycaemic T1D patients. Consistently, high glucose was shown to stimulate endothelial transport of dextran through the activation of the Rho signalling pathway, which leads to the contraction of the cytoskeleton and the consequent loss of endothelial connections [21]. We focused on the identification of the mediators involved in high glucose-induced hyperpermeability with the goal of individuating potential biochemical markers that might be useful for the early recognition of alterations of the endothelial barrier and, eventually, become a target to limit and delay atherogenesis. Among others, NO modulates endothelial cell permeability in vivo and in vitro [22], because it regulates cytoskeletal architecture through Rho [12] and downregulates VE-cadherin [22]. Therefore, we evaluated the levels of NOx in the sera from our T1D patients and found a very strong association between elevated amounts of serum NOx and fasting glucose concentrations. We argue that transient isolated peaks of glycaemia can be detrimental for the integrity of the endothelium because they increase permeability by stimulating NO production. This is particularly true in the light of the so-called “metabolic memory”, a theory indicating that glycaemic instability promotes metabolic and epigenetic changes that remain also when glucose levels return normal [23]. Indeed, exposure of HUVEC to oscillating high glucose is more detrimental than constant high glucose and induces a metabolic memory after glucose normalization [24]. It is, therefore, important to establish a therapeutic/dietetic regimen which keeps glycaemia within the physiological range.

While higher amounts of serum NOx in T1D patients versus controls are reported also by other authors [17,25], the source of NOx remains undetermined. T1D is associated with a pro-inflammatory environment [26,27]. Plasma concentrations of pro-inflammatory cytokines IL-1β and IL-17A, as well as T cell synthesized cytokines IFN-γ, TNF-α and IL-23, are increased in T1D subjects [27]. These cytokines activate iNOS and stimulate NO synthesis in various cell types, including the vascular cells. Accordingly, lung microvascular endothelial cells cultured in a high glucose-containing medium and then challenged with LPS upregulate iNOS [28]. However, no evidence is provided that the increased amounts of iNOS is responsible for hyperpermeability, since no pharmacological or genetic inhibition was performed [28]. Additionally, considering the high heterogeneity of the endothelium and the unique features of lung vasculature, human pulmonary microvascular endothelial cells are likely to behave differently from the macrovascular endothelial cells used in our experiments [29].

Our results point to a direct effect of high glucose in increasing NOx levels. Indeed, HUVEC cultured in media containing high glucose release more NOx than controls in a dose-dependent fashion through the upregulation of iNOS. Accordingly, genetic or pharmacological inhibition of iNOS prevents the increase of endothelial permeability caused by the sera from hyperglycaemic T1D patients. In agreement with our findings, in alloxan-induced T1D rats NOx are increased through the overexpression of iNOS [30]. To this purpose, it is interesting that aerobic exercise significantly decreases iNOS in pre-diabetic rats [31]. Therefore, physical activity might represent a preventive strategy to control iNOS expression in T1D patients, thus tempering endothelial damage. Moreover, it should be recalled that, besides NOS-derived NO, the steady-state systemic NO concentrations are regulated by the dietary intake of nitrates and nitrites that can be metabolized into bioactive NO via stepwise reductions [32]. Currently, no data are available about tailored nutritional approaches as tools to control NOx levels and prevent endothelial hyperpermeability in T1D patients [33]. 

A last point needs to be considered, i.e., the predictive value of measuring HbA1c as a marker to individuate early alterations of endothelial function. We found increased NOx amounts independently from HbA1c levels. Interestingly, increased levels of NOx were reported also in patients with T2D, the most prevalent form of diabetes. Different from our findings in T1D, NOx levels positively correlate with both HbA1c and fasting glycaemia in T2D individuals [17]. It would be interesting to unveil the effects of the sera from T2D patients on endothelial permeability and the levels of NOS.

To conclude, although limited by the small number of subjects included in the study, our results suggest that sporadic transitory peaks of glycaemia in T1D patients lead to the activation of iNOS. The consequent increase of NOx impairs the endothelial barrier, thus facilitating subendothelial accumulation of macromolecules that alter the microenvironment of the intima and promote vascular disease. 

## 4. Materials and Methods

### 4.1. Study Population

This is a cohort study in which each participant who fulfilled the inclusion criteria was consecutively enrolled. The study was carried out in 36 paediatric T1D patients (T1D) and in 14 age-matched non-diabetic healthy donors (CTR). Clinical characteristics of T1D patients and healthy controls are summarized in Table 1. None of the diabetic patients was affected by other complications, such as retinopathy (evaluated by stereoscopic fundus photography) or neuropathy (evaluated by nervous conduction velocity and autonomic tests) or was in treatment with other drugs except insulin. All patients included in the study were nonsmokers; none was taking antioxidant supplements or drugs with known antioxidant activity. Normal glycated haemoglobin (HbA1c) is between 4% and 7.5%. Normal glycaemia ranges between 60 and 100 mg/dL, while fasting hyperglycaemia was defined for values higher than 100 mg/dl. Informed consent was obtained from all subjects included in the study. Sera were collected at the University of Milan–V. Buzzi Children’s Hospital. The study was approved by the Buzzi Children’s Hospital (ASST Fatebenefratelli–Sacco, Milan, Italy) Ethical Committee (2018/ST/143, 9th October 2018, Milano Area 1). All procedures followed were in accordance with the ethical standards of the responsible committee on human experimentation (institutional and national) and with the Declaration of Helsinki 1975, as revised in 2008. 

### 4.2. Cell Culture 

Human Umbilical Vein Endothelial Cells (HUVEC) were obtained from the American Type Culture Collection (ATCC, Manassas, WV, USA) and cultured in medium M199 (Euroclone, Milano, Italy) with 10% fetal bovine serum (FBS), 1 mM L–Glutamine, 1 mM Sodium Pyruvate, 1 mM Penicillin–Streptomycin, 5 U/mL Heparin and 150 µg/mL Endothelial Cell Growth Factor on 2% gelatin-coated dishes [34] added. To test the sera of the participants, FBS was substituted by 10% of serum collected from subjects (CTR) or paediatric diabetic patients (T1D). The cells were routinely tested for the expression of endothelial markers and used for 6–7 passages. To perform the experiments, the cells were trypsinized, stained with 0.4% trypan blue solution and counted using a Luna Automated Cell Counter (Logos Biosystems, Anyang–si, Gyeonggi–do, South Korea). d–glucose (Sigma–Aldrich, St. Louis, MO, USA) was used at the concentrations of 11.1 mM and 30 mM and L–glucose (Sigma–Aldrich) was used as a control of osmolarity at the concentration of 30 mM.

Nitric Oxide Synthase (NOS) was inhibited using either small interfering RNA (siRNA) or pharmacological inhibitors. Subconfluent cells were transfected with siRNA targeting *eNOS* (NOS3) [20 nmol, 5′-TTCGAGGGACACCACGTCATACTCA-3′ (Invitrogen Corporation, Carlsbad, CA, USA)] or *iNOS* (NOS2A) [20 nmol, 5′-ATCGAATTTGTCAACCAATAT-3′ (Invitrogen)] [35]. Lipofectamine RNAiMAX was used as a transfection reagent (Invitrogen), according to the manufacturer’s recommendations. After 6 h, the siRNA transfection medium was replaced with a culture medium added with 11.1 mM or 30 mM of glucose. The same experimental approach was used with 10% serum collected from CTR or T1D patients. We tested *eNOS*- and *iNOS*-silencing using Real Time PCR (not shown). Alternatively, subconfluent cells were pre-treated with 100 μM of pharmacological inhibitors of eNOS [L-Nω–Nitroarginine-Methyl-Ester (L-NAME) and iNOS [N6–(1-Iminoethyl)-l-Lysine (L-NIL) (Sigma–Aldrich) for 1 h. Then, 10% of serum from the cohort or two different concentrations of glucose were added. The experiment lasted 24 h. All the experiments were performed in triplicate 3 times. 

### 4.3. Transwell Permeability Assay 

The Transwell Permeability Assay was performed in a 24-well receiver plate with individual hanging cell culture inserts (Transwell^®^ Permeable Supports, Euroclone, 0.4 µm micropores, (Euroclone, Milano, Italy)). HUVEC were seeded into the inserts and, when confluent, incubated for 24 h with a medium containing 10% of serum collected from CTR or T1D patients or with a culture medium containing 11.1 mM or 30 mM glucose. After the treatment, 1 mg/mL Fluorescein isothiocyanate labelled–albumin (FITC–BSA) (Sigma–Aldrich), a fluorescent probe able to cross the monolayer of endothelial cells at a rate proportional to the monolayer’s permeability, was added [36]. The extent of permeability was determined by measuring the fluorescence in the lower compartment. Fluorescence was detected by the Promega Glomax Multi Detection System at excitation and emission spectrum wavelengths of 495/519 nm. The experiment was performed in triplicate 2 times. 

### 4.4. NOS Activity

NOS activity was measured in the culture media and in the serum from the patients using the Griess Assay [19] which measures NO oxidative products (NOx). Briefly, culture media or sera were mixed with freshly prepared Griess reagent and the absorbance was measured at 550 nm. The concentration of nitrites in the samples was determined using a calibration curve generated with a known concentration of sodium nitrite (NaNO_2_) solutions. The experiment was performed in triplicate 3 times. 

### 4.5. Western Blot Analysis

HUVECs were lysed in 50 mM Tris–HCl (pH 7.4) containing 150 mM NaCl, 1% NP40, 0.25% sodium deoxycholate, protease inhibitors (10 µg/mL Leupeptin, 10 µg/mL Aprotinin, 1 mM PMSF) and phosphatase inhibitors (1 mM sodium fluoride, 1 mM sodium vanadate, 5 mM sodium phosphate). Lysates (40 µg/lane) were separated on SDS–PAGE and transferred to nitrocellulose sheets at 400 mA for 2 h at 4 °C. Western Blot analysis was performed using antibodies against iNOS (BD Biosciences, Milano, Italy), P-eNOS^Ser1177^ (Cell Signaling Technology, Danvers, Massachusetts, USA), eNOS (BD Biosciences) and Actin (Santa Cruz Biotechnology, Dallas, TX, USA) [37]. After extensive washing, secondary antibodies labelled with horseradish peroxidase (GE Healthcare, Waukesha, WI, USA) were used. Immunoreactive proteins were detected by the SuperSignal Chemiluminescence Kit (Thermo Fisher Scientific Waltham, MA, USA) [35]. The experiment was performed 3 times and quantified using Image J software (National Institutes of Health, Bethesda, MD, USA).

### 4.6. Statistical Analysis 

Data are reported as means ± SD. The data were normally distributed and they were analyzed using one-way repeated measures ANOVA. The *p*-values deriving from multiple pairwise comparisons were corrected by the Bonferroni method. Statistical significance was defined for *p*-value ≤ 0.05. Regarding the Figures, * *p* ≤ 0.05; ** *p* ≤ 0.01; *** *p* ≤ 0.001. 

## 5. Conclusions

In this study, we show that sera from hyperglycaemic T1D patients significantly increase endothelial permeability through the upregulation of iNOS. Therefore, the identification of iNOS as a possible biomarker that promotes the insurgence of vascular disease in T1D could entail potential planning for the prevention of cardiovascular complications in T1D.

## Figures and Tables

**Table 1:** Clinical characteristics of paediatric T1D patients and healthy controls. Among the T1D patients, 10 were normoglycaemic, 26 hyper-glycaemic, 11 had high levels of HbA1c (>7.5 %) and 25 had levels of HbA1c within the physiological range.

	Healthy Subjects (*n* = 14)	Patients (*n* = 36)
**Sex**		
Male	*n* = 5	*n* = 12
Female	*n* = 9	*n* = 24
**Age (years)**		
Mean	12.5	13.8
Range	3.1–23.7	4.0–24.0
**Hb1Ac (%)**		
Mean	5.7	7.15
Range	5.30–6.80	5.10–8.80
**PCR (mg/dL)**		
Mean	0.53	0.71
Range	0.43–0.61	0.20–2.60
**Glycaemia (mg/dL)**		
Mean	102	168.26
Range	89.0–117.0	40.0–350.0
**Therapy**	None	MDI (*n* = 22) CSII (*n* = 14)

## Abbreviations

T1D	Type 1 diabetes
NO	Nitric Oxide
ROS	Reactive Oxygen Species
eNOS	endothelial Nitric Oxide Synthase
eNOSSer1177	eNOS phosphorylated on Ser1177
iNOS	inducible Nitric Oxide Synthase
NOx	nitrate and nitrite
Hb1Ac	glycated haemoglobin
HUVEC	Human Umbilical Vein Endothelial Cells
FBS	Fetal Bovine Serum
L-NAME	L-Nω–Nitroarginine-Methyl-Ester
L-NIL	N6–(1-Iminoethyl)-L-Lysine
LPS	lipopolysaccharide
